# Development and validation of identification models for aortic dissection and non-ST-segment elevation acute coronary syndrome in the emergency department

**DOI:** 10.1038/s41598-025-31275-9

**Published:** 2025-12-14

**Authors:** Yaxin Ban, Hao Wang, Yunfei Gu, Haojie Chen, Rubing Liang, Yuge Jin, Zhishuai Li, Xingke Li, Songsen Li

**Affiliations:** 1 Department of Cardiology, Luoyang Central Hospital Affiliated to Zhengzhou University, Luoyang, 471000 China; 2 Luoyang Maternal and Child Health Hospital, No. 206 Tongqu Road, Luoyang, 471000 China; 3 Department of Cardiology, First People’s Hospital of Xinxiang City, Xinxiang, 453000 China

**Keywords:** Cardiology, Computational biology and bioinformatics, Diseases, Health care, Medical research, Risk factors

## Abstract

Aortic dissection (AD) and non-ST-segment elevation acute coronary syndrome (NSTE-ACS) are critical illnesses whose prompt identification within the emergency department is challenging. This study aimed to establish rapid discrimination models to differentiate between these conditions. Patients of the training set and validation set were collected from January 2020 to June 2023. All patients used their final diagnosis. Discriminant models were constructed via univariate and multivariate logistic regression analyses. Based on the results of the two models, two web calculators were developed. A total of 1314 patients were included in the study, with 997 patients (399 AD patients and 598 NSTE-ACS patients) and 317 patients (132 AD patients and 185 NSTE-ACS patients) in the training and validation sets, respectively. The semi-model consisted of six clinical characteristics (age, heart rate, pulse pressure, temperature, hypertension, diabetes), with an area under the ROC curve (AUC) of 0.792 and 0.823 in the training and validation sets. The whole-model included five clinical characteristics (age, pulse pressure, hypertension, diabetes) and two point-of-care test data (high sensitivity troponin I, D-dimer). It had a higher predictive value compared to the semi-model, with AUCs of 0.973 and 0.980 in the training and validation sets, respectively. Given the optimal cutoff point, the semi-model demonstrated a sensitivity of 0.716 and a specificity of 0.734, whereas the whole-model displayed a sensitivity of 0.930 and a specificity of 0.946. Both identification models can be used as reliable tools for rapidly identifying AD and NSTE-ACS.

**Supplementary Information:**

The online version contains supplementary material available at 10.1038/s41598-025-31275-9.

## Introduction

Aortic dissection (AD) and acute coronary syndrome (ACS) are highly perilous, acute-onset, rapidly progressive conditions commonly encountered in chest pain centers^1,2^. Clinical manifestations include chest pain, and the accompanying symptoms lack specificity. A meta-analysis revealed a misdiagnosis rate of 33.8% (562 out of 1663 cases) for AD, with ACS being the most mistaken diagnosis^3^. To prevent the risk of dissection rupture and bleeding, active preoperative preparation must be provided and unnecessary transportation for patients with AD must be avoided^4^. Notably, the standard treatment for ACS, which involves early dual antiplatelet therapy^5,6^, can be fatal for AD patients and may increase the risk of interlayer rupture^7^. Therefore, promptly and accurately differentiating between the two conditions is essential, especially for non-ST-segment elevation (NSTE-ACS), which can be challenging to distinguish from AD given non-specific electrocardiographic manifestations. Unfortunately, no efficient identification models exist for AD and NSTE-ACS in emergency departments.

Therefore, based on the TRIPOD statement^8^, this study collected clinical characteristics and point-of-care test (POCT) data from patients in a chest pain center and developed semi-model (i.e., including only clinical characteristics) and whole-model (i.e., including clinical characteristics and POCT data) for patients with AD and NSTE-ACS. Decision curves were used to assess the clinical benefits^9,10^, and user-friendly web calculators were developed to assist in rapid clinical diagnosis.

## Methods

### Study patients and protocol

We collected data retrospectively from patients with AD and NSTE-ACS at the Chest Pain Center of Luoyang Central Hospital between Jan 2020 and Jun 2022 for the training set, and prospectively collected the data from Jul 2022 to Jun 2023 for the validation set. All patients used their final diagnosis. The prediction model intends to utilize up to eight candidate predictors, with a C statistic of 0.75. The ratio of AD patients to those with NSTE-ACS in the chest pain center, as determined from the preliminary experiment, is 2:3. Consequently, the final calculated minimum sample size required for this study is 590 cases, and the sample size for the training set meets the established standards. The AD cohort included patients with both Stanford Type A and Type B aortic dissections, confirmed by computed tomography angiography (CTA). The diagnostic criteria for NSTE-ACS conform to the 2020 guidelines of the European Society of Cardiology for managing patients experiencing ACS in the absence of sustained ST-segment elevation. These criteria have been verified through coronary angiography (CAG). Exclusion criteria included: (1) Severe infection. (2) Severe liver and kidney dysfunction (Child-Pugh grade B or C, decompensated renal function, or dialysis use), malignant tumors. (3) Long-term anticoagulant drug use. (4) Refusal of CTA and CAG examinations. This study was approved by the Ethics Committee of Luoyang Central Hospital Affiliated to Zhengzhou University, and complied with the Declaration of Helsinki. All data were analyzed anonymously. Patients in the prospective cohort provided written informed consent before being included in the study. For the retrospectively collected data used in this study, patient informed consent was waived by the Ethics Committee due to the retrospective nature of the study and the use of anonymized data.

### Collection of data

Demographic data, clinical features, medical history, and laboratory data—from POCT equipment—were collected from all patients during their first medical contact. Demographic information included gender and age. Clinical features included respiration, heart rate, blood pressure, and body temperature; mean arterial pressure (MAP) and pulse pressure were calculated. Medical history included hypertension, diabetes, and ischemic stroke, The diagnosis of ischemic stroke, based on patient history, involves clinical assessment and imaging confirmation (MRI). Diagnostic criteria corresponded to relevant guidelines^11–13^. Laboratory data, including D-dimer and high sensitivity troponin I (HsTnI), were obtained through POCT equipment. They were drawn at the time of the first blood draw in the emergency department, immediately upon arrival. All data were double-entered and compared using Epidata (version 3.1).

### Statistical analysis

**Fig. 1:**
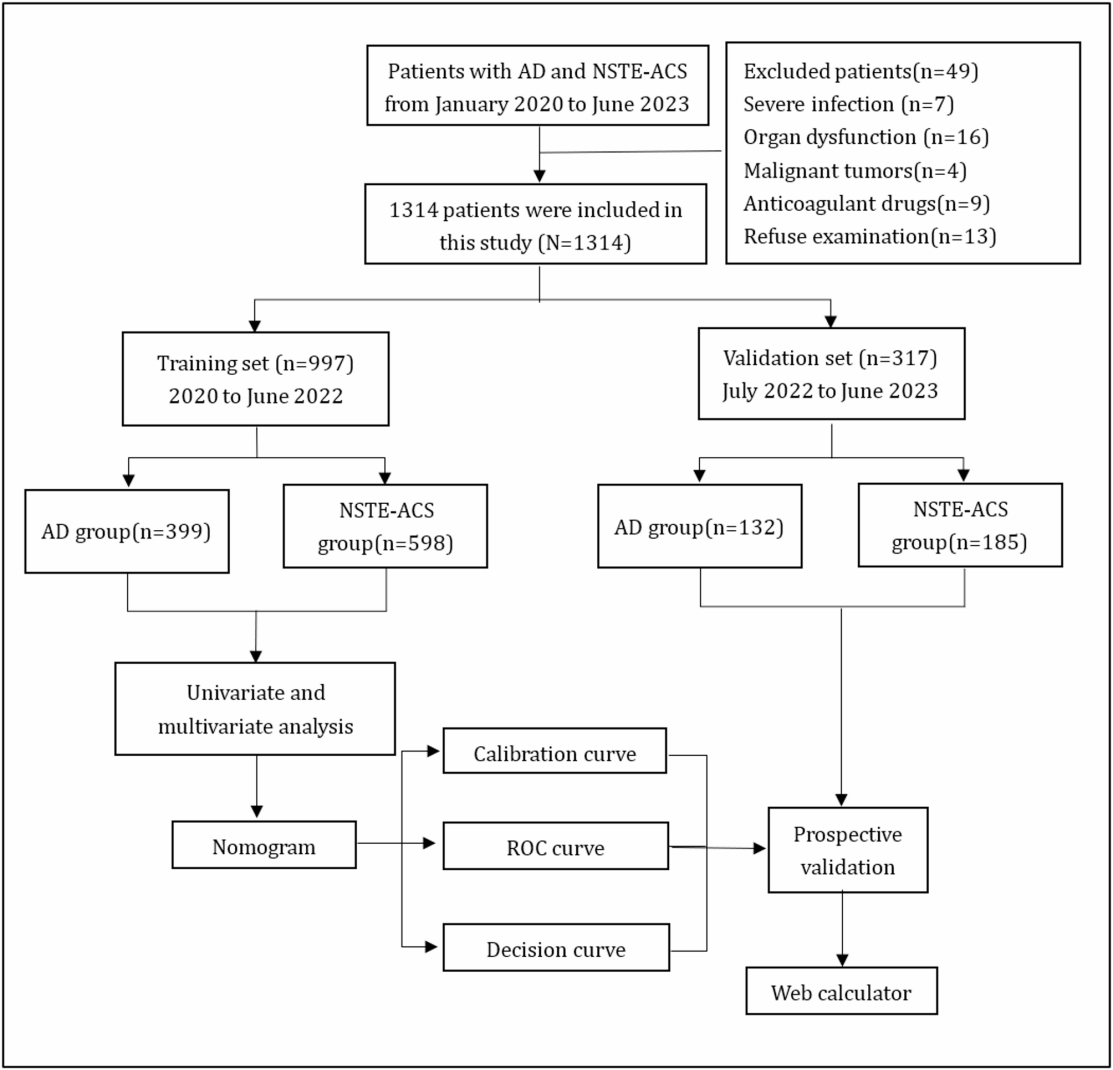
Flow chart of the research. AD: Aortic dissection; NSTE-ACS: non-ST-segment elevation acute coronary syndrome; ROC: receiver operating characteristic.

Figure 1 demonstrates the overall design of the experiment. In the training set, both the semi-model and the whole-model were constructed, and subsequently verified in the verification set. The Shapiro–Wilk test was used to analyze the normality of continuous variables. Mean ± standard deviation represented variables with a normal distribution. To evaluate the uniformity of variances, Levene’s test was used. When variances were equivalent, the Student’s t-test was applied, whereas the Welch’s t-test was utilized in cases with unequal variances. For variables that deviated from a normal distribution, medians and inter quartile ranges (IQR) were employed, and group disparities were assessed through the Mann-Whitney U test. For categorical variables, the chi-square test was employed to analyze differences between groups. All hypothesis tests were two-sided and assumed a significance level of 0.05.

### Construction and validation of identification models

To perform missing value imputation, multiple imputations using R’s mice package were employed with the “pmm” interpolation method and a seed of 999 was set. The variable with the highest missing proportion was HsTnI, with a missing proportion of 30%. Therefore, 30 interpolations were performed^14^, a final complete dataset for subsequent analysis was obtained by pooling these datasets according to Rubin’s rules. To evaluate the presence of multicollinearity, the car package was utilized to calculate the variance inflation factor (VIF), excluding variables showing high multicollinearity. Variables were retained for the multivariate logistic regression analysis to construct a predictive model with a P value less than 0.1. Then odds ratios (OR) and 95% confidence intervals (95%CI) were calculated. Subsequently, leveraging the findings from the multi-factor logistic regression analysis, nomograms were created to visually represent the probability of individuals experiencing AD.

To assess the calibration and discrimination of the two models, calibration and receiver operating characteristic (ROC) curves were generated in the training set. To evaluate the calibration curve, bootstrap analysis with 1000 repetitions was employed, while the ROC curve relied on the predicted scores from the nomogram. Subsequently, the area under the ROC curve (AUC) was calculated. The same methods were applied to the verification set. Decision curves were then plotted with the training and verification sets to evaluate the clinical net benefits of the models. To improve accessibility, a user-friendly web calculator was developed utilizing the Shiny package and platform. The calculator integrated the results from the two models, providing an interface for users to easily access the outcomes. All statistical analyses were performed using R software (version 4.2.3). The software packages used included mice, reportReg, rms, car, pROC, dcurves, DynNom, and Shiny.

## Results

### Baseline characteristics of patients

1314 participants were enrolled, with 997 and 317 individuals in the training and validation sets, respectively. Table 1 describes patient demographic information, clinical characteristics, medical background, and laboratory findings for both sets. Within the training set, the AD group exhibited higher levels of body temperature, D-dimer and greater frequencies of male patients and hypertension compared to the NSTE-ACS group. Conversely, the AD group displayed lower levels of HsTnI, young ages and fewer frequencies of diabetes and stroke history compared to the NSTE-ACS group. Heart rate, pulse pressure, systolic blood pressure, and MAP had no noticeable disparities between the two groups.

**Table 1:** Baseline characteristics of Patients.

Characteristics	Training group(*n* = 997)			Validation group(*n* = 317)		
	AD(*n* = 399)	NSTE-ACS (*n* = 598)	*P* value	AD(*n* = 132)	NSTE-ACS (*n* = 185)	*P* value
Demographic data						
Male sex, n (%)	311(77.94)	407(68.06)	0.001	111(84.09)	133(71.89)	0.02
Age (years)	53(44–62)	64(55–73)	< 0.001	55(46–63)	65(55–72)	< 0.001
Clinical features						
Respiration rate (/min)	20(18–20)	19(18–20)	0.06	20(18–20)	19(19–20)	0.07
Heart rate (/min)	74(66–84)	75(66–86)	0.22	78(66–86)	78(70–84)	0.62
SBP (mmHg)	140(120-161.5)	140(126–160)	0.767	150(125–176)	136(120–154)	< 0.001
Pulse pressure (mmHg)	59(47–70)	55(48–70)	0.14	68(51–80)	55(47–66)	< 0.001
MAP (mmHg)	102(87-118.5)	103(93–117)	0.23	103(90–123)	99(89–108)	0.02
Body temperature (℃)	36.5(36.2–36.6)	36.4(36.2–36.5)	0.008	36.5(36.5–36.7)	36.5(36.3–36.6)	< 0.001
Medical history						
Hypertension, n (%)	297(74.44)	365(61.04)	< 0.001	109(82.58)	105(56.76)	< 0.001
Diabetes, n (%)	13(3.26)	165(27.59)	< 0.001	2(1.52)	55(29.73)	< 0.001
Stroke, n (%)	30(7.52)	96(16.05)	< 0.001	10(7.58)	30(16.22)	0.04
Laboratory data						
HsTnI (ng/mL)	0.032(0.005–0.138)	1.701(0.139–6.840)	< 0.001	0.006(0.001–0.730)	0.514(0.034–5.029)	< 0.001
D-dimer (ng/mL)	3093(1751–4745)	140(80–310)	< 0.001	3920(2274–7620)	190(160–220)	< 0.001

AD: Aortic dissection; NSTE-ACS: non-ST-segment elevation acute coronary syndrome; SBP: Systolic blood pressure; MAP: Mean arterial pressure; HsTnI: high sensitivity troponin I.

### Feature selection

After performing the VIF calculation, multicollinearity was noted among systolic blood pressure, pulse pressure difference, and MAP. By excluding variables separately, all variables achieved the minimum VIF value (with the maximum VIF value being 1.46) when systolic blood pressure was excluded. Univariate logistic regression analysis was conducted. The outcome is presented in Table 2. To construct the whole-model, a multivariate logistic regression analysis was performed by incorporating variables with P-values < 0.1. After excluding laboratory data, the remaining variables were used in the regression analysis to create the semi-model. The OR and 95%CI for both models are presented in Table 3.

**Table 2:** Univariate logistic regression analysis of variables estimated by using the data from the training set.

Variables	OR (95% CI)	*P* value
Male sex	1.659(1.238–2.222)	0.001
Age	0.940(0.930–0.951)	< 0.001
Respiration	1.041(0.979–1.107)	0.20
Heart rate	0.992(0.984-1.000)	0.06
Pulse pressure	1.006(0.999–1.013)	0.08
MAP	0.998(0.991–1.004)	0.49
Body temperature	2.186(1.315–3.633)	0.003
Hypertension	1.859(1.407–2.456)	< 0.001
Diabetes	0.088(0.049–0.158)	< 0.001
Stroke	0.425(0.276–0.654)	< 0.001
HsTnI	0.675(0.618–0.737)	< 0.001
D-dimer	1.002(1.001–1.002)	< 0.001

OR: odds ratio; MAP: mean arterial pressure; HsTnI: high sensitivity troponin I.

**Table 3:** Multivariate logistic regression analysis of variables estimated by using the data from the training set.

Variables	Semi-model		Whole-model	
	OR (95% CI)	*P* value	OR (95% CI)	*P* value
Male sex	0.919(0.645–1.309)	0.64	1.451(0.806–2.612)	0.214
Age	0.943(0.931–0.955)	< 0.001	0.934(0.914–0.953)	< 0.001
Heart rate	0.987(0.977–0.997)	0.013	0.986(0.969–1.003)	0.095
Pulse pressure	1.009(1.001–1.017)	0.04	1.017(1.004–1.031)	0.011
Body temperature	2.052(1.139–3.696)	0.02	1.258(0.466-3.400)	0.65
Hypertension	2.539(1.828–3.525)	< 0.001	3.015(1.699–5.349)	< 0.001
Diabetes	0.094(0.052–0.173)	< 0.001	0.115(0.048–0.275)	< 0.001
Stroke	0.612(0.374–1.002)	0.051	0.706(0.323–1.547)	0.385
HsTnI	-	-	0.819(0.757–0.886)	< 0.001
D-dimer	-	-	1.002(1.001–1.002)	< 0.001

OR: odds ratio; HsTnI: high sensitivity troponin I.

### Construction and verification of the semi-model for AD and NSTE-ACS

**Fig. 2:**
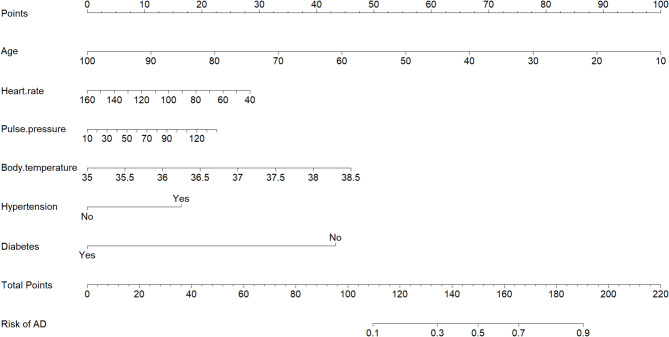
Nomogram for the semi-model. AD: Aortic dissection; HsTnI: high sensitivity troponin I.

**Fig. 3:**
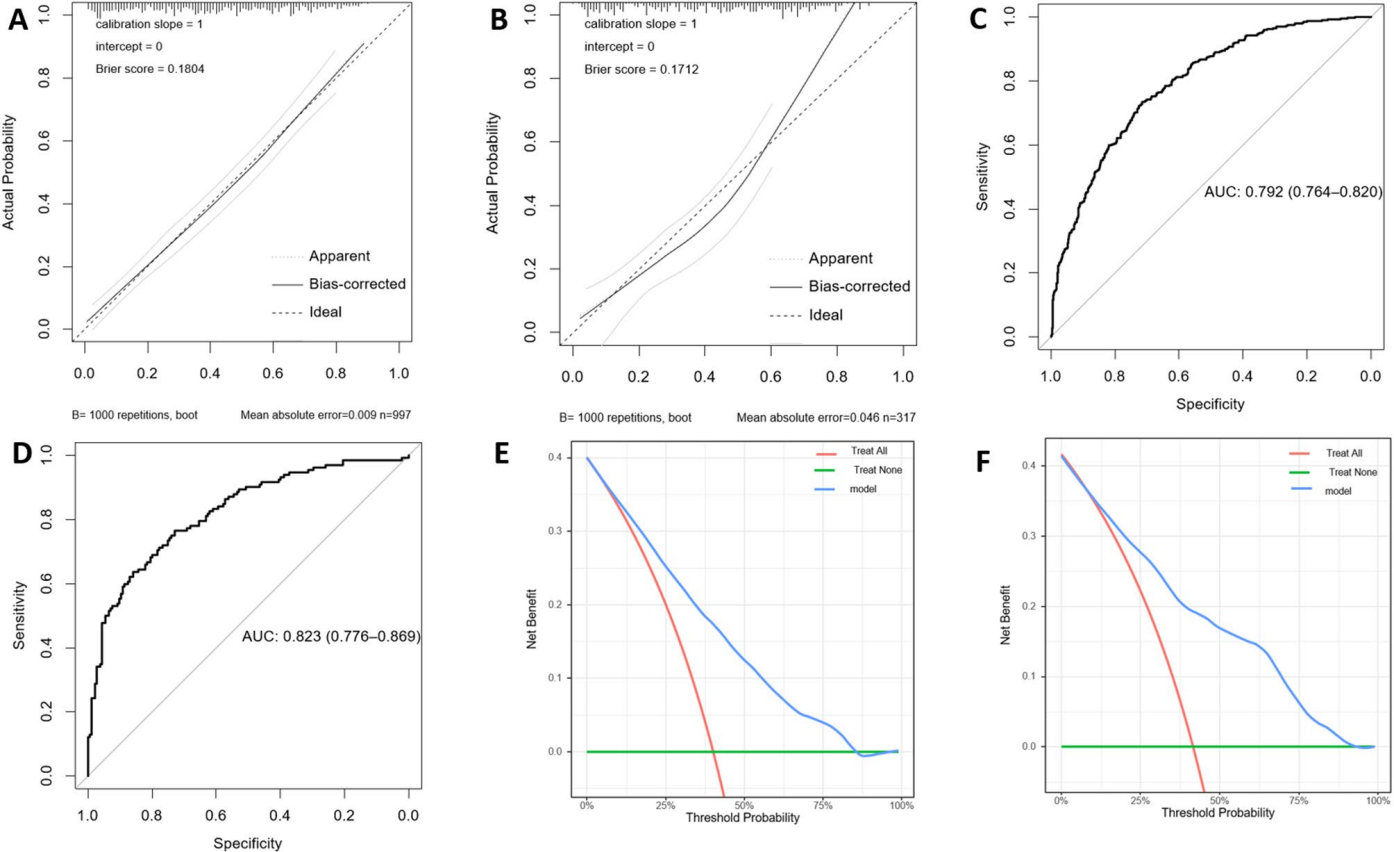
Assessment of the semi-model. Calibration plots in the training set (**A**) and validation set (**B**); ROC curves in the training set (**C**) and validation set (**D**); Decision curves in the training set (**E**) and validation set (**F**); AUC: area under the receiver operating characteristic curve; ROC: receiver operating characteristic.

A nomogram was developed using the semi-model to predict the probability of AD based on six clinical characteristics (Fig. 2). The nomogram was based on independent predictors identified in the training set. Each clinical characteristic was assigned a score ranging from 1 to 100. Individual scores were combined for the total score, which was used to estimate the probability of AD. The predicted probability aligned well with the actual occurrence probability of AD, as demonstrated by the calibration curve (Fig. 3A, B). The ROC curve (Fig. 3C, D) showed that the AUC in the training set was 0.792 (95% CI: 0.764–0.820), with a score cut-off of 143.6 proving most effective. At the optimal threshold, the sensitivity and specificity were 0.716 and 0.734, the positive predictive value (PPV) and the negative predictive value (NPV) were 0.633 and 0.801. The AUC was 0.823 (95%CI: 0.776–0.869) in the validation set. The decision curve (Fig. 3E, F) showed that the semi-model exhibited good net benefit when the threshold probability ranged from 10% to 85%. A webpage for the semi-model was developed using the Shiny package and platform (https://banyx.shinyapps.io/semi_model/).

### Construction and validation of the whole-model for AD and NSTE-ACS

**Fig. 4:**
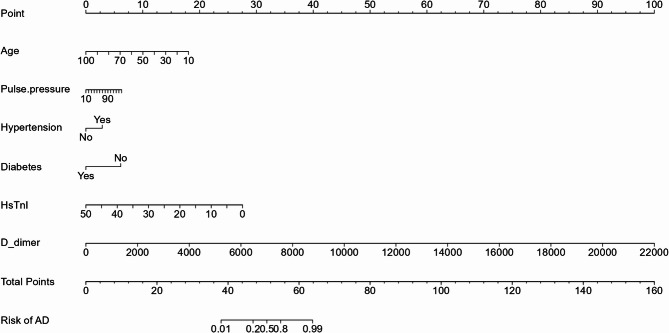
Nomogram for the whole-model. AD: Aortic dissection. HsTnI: high sensitivity troponin I.

**Fig. 5:**
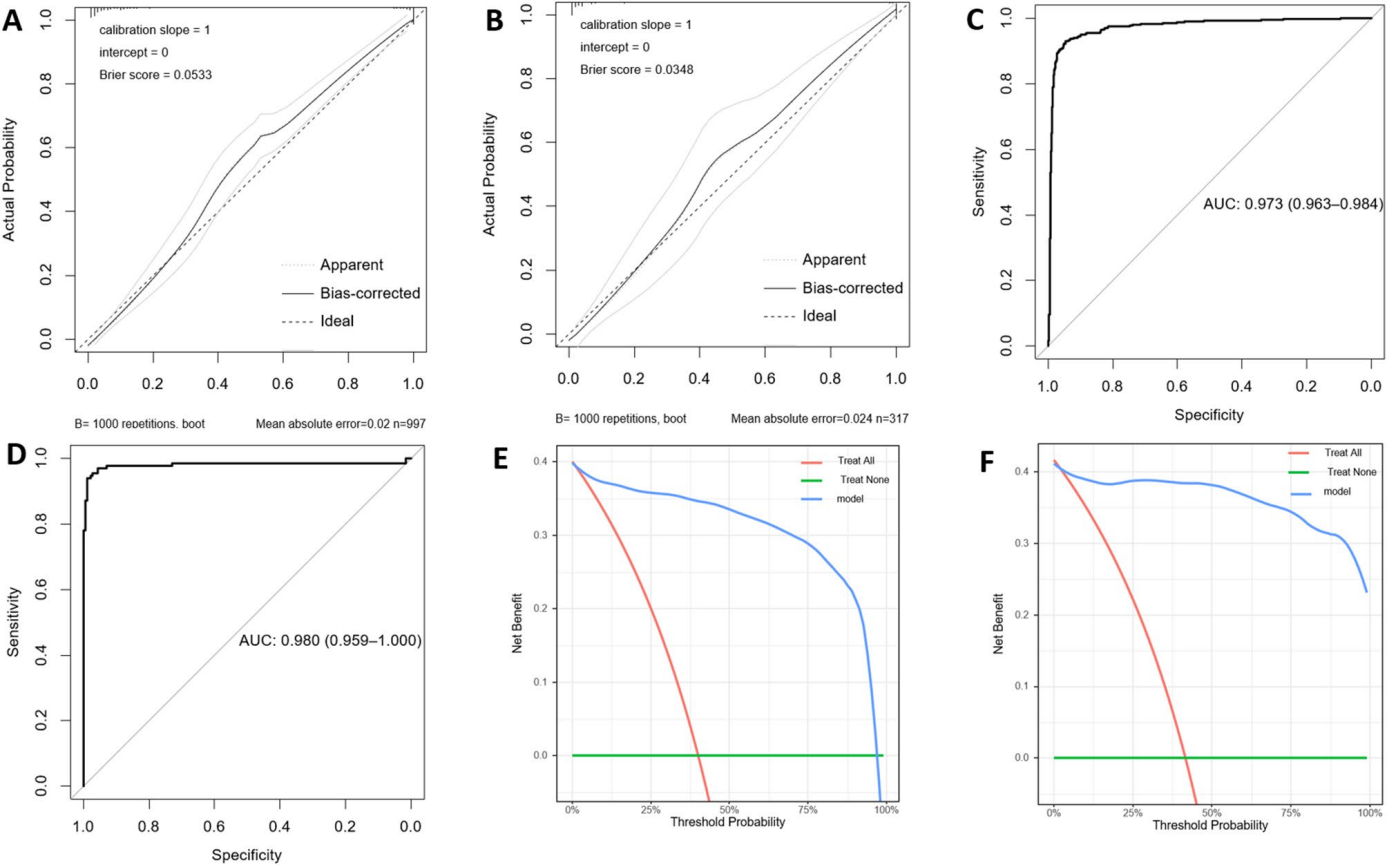
Assessment of the whole-model. Calibration plots in the training set (**A**) and validation set (**B**); ROC curves in the training set (**C**) and validation set (**D**); Decision curves in the training set (**E**) and validation set (**F**); AUC: area under the curve; ROC: receiver operating characteristic; ROC: receiver operating characteristic.

A nomogram was developed using the whole-model to predict the probability of AD based on six clinical characteristics. It incorporated five clinical characteristics and two laboratory test outcomes (Fig. 4); its accuracy was verified using the same method. The calibration curve (Fig. 5A, B) displayed a close alignment between the predicted likelihood and the actual occurrence rate of AD. Moreover, the calibration curve results from the validation set surpassed those of the semi-model. The AUC was 0.973 (95%CI: 0.963–0.984) in the training set, with a score cut-off of 49.853 proving most effective. At this threshold, the sensitivity and specificity were 0.930 and 0.946, the PPV and the NPV were 0.9213 and 0.953. The AUC was 0.980 (95%CI: 0.959–1.000) in the validation set, indicating superior performance compared to the semi-model (Fig. 4C, D). The decision curves (Fig. 5E, F) illustrated that the whole-model consistently achieved better net benefit across all threshold probabilities. Finally, a web page for the whole-model (https://banyx.shinyapps.io/whole_model/) was also developed.

## Discussion

This study developed and validated two identification models to differentiate between AD and NSTE-ACS. The semi-model was based on six clinical characteristics: age, heart rate, pulse pressure, body temperature, hypertension, and diabetes. Meanwhile, the whole-model, which incorporated laboratory test results, consisted of five clinical characteristics and two laboratory parameters: age, heart rate, pulse pressure, hypertension, diabetes, HsTnI and D-dimer. The models were prospectively verified, and ROC, calibration, and decision curves were plotted in training and validation sets. The outcomes demonstrated that both models exhibited good discrimination, calibration, and clinical net benefit. Moreover, the whole-model, showed a higher predictive value compared to the semi-model.

Clinical prediction models, as common clinical tools, are noteworthy in the diagnoses and treatment of diseases^8^. However, few identification models exist for AD and NSTE-ACS. In a study^15^, involving 263 patients, 94 patients were diagnosed with AD, while the rest presented with NSTE-ACS. Training and validation sets were created following a 3:1 ratio. This study developed a model that identified four significant predictors and two clinically significant factors. While pain level and tearing pain in the model may vary based on patient tolerance, introducing subjective bias. In another investigation^2^, a grand sum of 638 participants were divided into two groups: with chest pain less than 2 h and between 2 and 3 h. Prediction models were built for each group. However, the time window of this model was limited, making it challenging for patients to accurately determine the exact onset of the disease. This time constraint also posed difficulties for medical personnel in selecting the appropriate model. Moreover, bilirubin levels used in the model cannot be obtained through POCT. Considering these factors, further validation is required before applying these models in the emergency department.

In individuals experiencing AD, systolic hypertension aggravates the hemodynamic forces impacting the movable aortic arch and the stationary ascending and descending thoracic aortas^16^. Consequently, patients with AD have a higher pulse pressure. Although hypertension is also a recognized traditional risk factor for ACS, it may exert a more significant influence on the development of AD. Hoff analyzed data from the International Acute Aortic Dissection Registry and found that half of the patients with AD exhibited elevated pulse pressure^17^. This finding corroborates the results of the present study regarding elevated pulse pressure within the AD cohort. The study by Shimada suggested that the presence of a false lumen, progression of dissection, recurrence of dissection, or intramural thrombus may contribute to fever in patients with AD^18^. Inoue found that fever in AD patients could be associated with a systemic inflammatory response triggered by endogenous pro-inflammatory mediators within the body, which also affects the extrinsic coagulation pathway^19^. Zhang reported a significant increase in neutrophils among AD patients^20^, further indicating the likelihood of a systemic inflammatory response in this population. These studies support the notion that body temperature elevation is prevalent among patients with AD. Of note, the variable of body temperature was not included in the whole model. The statistically significant difference in body temperature between the two groups observed in this study might be a case of significance driven by the large sample size, given that the actual difference was merely 0.1 °C. In our study, the faster heart rate observed in NSTE-ACS patients may be attributed to sympathetic nerve excitement. However, as heart rate was not included in the full model, its role in discriminating between the two groups requires further investigation. Lastly, advanced age and diabetes, which are traditional risk factors for ACS^21^, align with the findings of this study. These findings supported the results of the semi-model.

Biomarkers helpful diagnose AD and differentiate it from other diseases that manifest with chest pain^20^. POCT enables the rapid detection of patient-related biomarkers at the bedside, thus benefiting patients, medical centers, and medical systems^22^. Currently, D-dimer and troponin are the most frequently reported biomarkers in identifying AD and NSTE-ACS; both are detectable using POCT equipment^23^. D-dimer is the degraded fragment of plasma fibrin after thrombus fibrinolysis^24^. It participates in and is used as a serum marker for coagulation and fibrinolysis^25^. In patients with AD, the interaction between blood and a non-endothelial false lumen triggers a series of coagulation and fibrinolytic reactions, increasing D-dimer levels. Numerous studies have confirmed this relationship^26–28^, and guidelines recommend considering elevated D-dimer as a diagnostic criterion for AD^29,30^. Nonetheless, certain investigations had also noticed heightened levels of D-dimer in individuals diagnosed with NSTE-ACS, albeit to a lesser degree than those with AD^31^. Additionally, patients with thrombosis also exhibited significantly elevated D-dimer levels^32^. These complicated the differentiation of D-dimer between AD and NSTE-ACS. Troponin I, a biomarker of myocardial damage, was recommended in 2000 for the diagnosis of acute myocardial infarction^33^. However, several hours are required for elevated troponins to be detected in the plasma after the disease onset^34,35^. Additionally, the level of troponin I can be normal in patients with unstable angina^36^. The more sensitive HsTnI can be detected in plasma within one to two hours of onset^37^ but lacks specificity and can also be elevated in some AD patients^4,38,39^. These limitations hinder the ability of HsTnI to differentiate between the two diseases. Therefore, the current study built a whole-model, which was more effective in identifying AD and NSTE-ACS, based on the semi-model by incorporating the above biomarkers that can be obtained through POCT.

One advantage of the current study is that the identification model was constructed using patient data from the chest pain center, which provides a more representative sample of the population. It also had a large sample size and collected easily obtainable data. Additionally, prospective validation was conducted. The semi-model can quickly generate results, allowing for initial assessment, or guide emergency treatment before transferring patients from primary to higher-level hospitals. The whole-model can be utilized in hospitals equipped with POCT equipment to guide the management of patients with acute chest pain. Furthermore, based on these two models, we have also applied for computer software copyright (Register number: 2024SR0061185) and created a WeChat mini program called AD and non-ST-segment elevation ACS probability calculator (AppID: wx6f3d1a76133d2208), allowing clinicians and individuals to quickly calculate the probability of suspected patients suffering from AD on their cell phones or computers. This tool is valuable for identifying clinical acute chest pain patients and providing timely emergency treatment.

### Limitations

Certain constraints are inherent to this research. First, the study was conducted in a specialized chest pain center within a single tertiary hospital, which may limit the generalizability of our findings to general emergency department populations, The potential for selection bias must be considered. Second, a significant limitation was the high proportion of missing data (30%) for the key predictor HsTnI in the training set. Although this was addressed using multiple imputation, this level of missingness introduces a degree of uncertainty into the model’s coefficients and performance that cannot be entirely eliminated. Third, this study did not document the time between symptom onset and blood collection. A difference in this interval between the two groups could potentially lead to bias in the experimental results. Finally, this investigation relied on data from a single center. While both models demonstrate promising clinical value in prospective verification, further validation of external applicability in other centers is required.

## Conclusion

In conclusion, this study developed and validated two identification models for distinguishing between AD and NSTE-ACS. These models can serve as effective tools for rapidly identifying the two conditions in standard chest pain centers and primary hospitals.

## Supplementary Information

Below is the link to the electronic supplementary material.

Supplementary Material 1

Supplementary Material 2

Supplementary Material 3

## Data Availability

Data are available from the corresponding author upon reasonable request.
