# Supplementary material for: Development and validation of identification models for aortic dissection and non-ST-segment elevation acute coronary syndrome in the emergency department
Source: Sci Rep. 2025 Dec 14;16:1693. doi: 10.1038/s41598-025-31275-9 (PMC12800131; doi:10.1038/s41598-025-31275-9)
Supplement: Supplementary file 1 — Supplementary Material 1 [file 41598_2025_31275_MOESM1_ESM.docx]

Semi_model:P=1/[1+exp(-(-0.0605×Age-0.0129×Heart.rate+0.0095×Pulse.pressure+0.7149×Body.temperature+0.8905×Hypertensin-2.3582×Diabetes-22.8113))]

The best cut-off : -0.345

Whole_model:P=1/[1+exp(-(-0.071Age+0.017×Pulse.pressure+1.0228×Hypertension-2.1838×Diabetes-0.1965×HsTnI+0.0016×D_dimer+0.8275))]

The best cut-off : -0.677
